# Incorrect sleeping position and eye rubbing in patients with unilateral or highly asymmetric keratoconus: a case-control study

**DOI:** 10.1007/s00417-020-04771-z

**Published:** 2020-06-10

**Authors:** Adrien Mazharian, Christophe Panthier, Romain Courtin, Camille Jung, Radhika Rampat, Alain Saad, Damien Gatinel

**Affiliations:** 1 Ophthalmology department, Fondation Ophtalmologique Adolphe de Rothschild, 25 Rue Manin, 75019 Paris, France; 2 Institut Laser Vision Noémie de Rothschild, Paris, France; 3 Clinical Research Center, Biological Resources Center, Centre Hospitalier Intercommunal de Créteil, Créteil, France; 4 Department of Ophthalmology, American University of Beirut – Medical Center, Beirut, Lebanon

**Keywords:** Eye rubbing, Unilateral keratoconus, Asymmetric keratoconus, Sleeping position, Risk factors, Case-control

## Abstract

**Purpose:**

To evaluate eye rubbing and sleeping position in patients with Unilateral or Highly Asymmetric Keratoconus (UHAKC).

**Methods:**

Case-control study of consecutive UHAKC patients diagnosed at the Rothschild Foundation. Controls were age- and sex-matched, randomly selected refractive surgery clinic patients. Patients self-administered questionnaires regarding their family history of keratoconus, eye rubbing, and sleeping habits. All the eyes underwent a comprehensive ocular examination. Logistic regression was used to analyze univariate and multivariate data to identify risk factors for keratoconus.

**Results:**

Thirty-three UHAKC patients and 64 controls were included. Univariate analyses showed that daytime eye rubbing [OR = 172.78], in the morning [OR = 24.3], or in eyes with the steepest keratometry [OR = 21.7] were significantly different between groups. Allergy [OR = 2.94], red eyes in the morning [OR = 6.36], and sleeping on stomach/sides [OR = 14.31] or on the same side as the steepest keratometry [OR = 94.72] were also significantly different. The multivariate model also showed statistical significance for most factors including daytime eye rubbing [OR = 134.96], in the morning [OR = 24.86], in the steepest eye [OR = 27.56], and sleeping on stomach/sides [OR = 65.02] or on the steepest side [OR = 144.02]. A univariate analysis in UHAKC group, comparing the worse and better eye, showed that eye rubbing [OR = 162.14] and sleeping position [OR = 99.74] were significantly (*p* < 0.001) associated with the worse eye.

**Conclusion:**

Our data suggests that vigorous eye rubbing and incorrect sleeping position are associated with UHAKC. This is especially true in rubbing the most afflicted eye, and contributory sleep position, including positions placing pressure on the eye with the steepest keratometry.

**Electronic supplementary material:**

The online version of this article (10.1007/s00417-020-04771-z) contains supplementary material, which is available to authorized users.

## Introduction

Keratoconus (KC) is a corneal ectatic disorder that has been classically defined as a progressive, asymmetric, and often bilateral, non-inflammatory condition occurring in adolescence or early adulthood that produces a thinning and steepening of the cornea and causes irregular astigmatism, myopia, and poor visual acuity [1]. The estimated prevalence of KC is approximately 50 to 230/100,000.^1^ People are usually affected bilaterally although it is often asymmetric [2]. The reported prevalence of eye rubbing ranges from 66 to 73% in patients with KC, which is concerning as approximately 15% of the worldwide population appears to be suffering from ocular allergies, even more so in industrialized countries [3]. Some studies support that KC is associated with parental consanguinity [4] or other genetic systemic disorders [5], which may render some population genetically more susceptible to the disease than others. Keratoconus could then be triggered by an environmental factor such as repeated biochemical stress on a genetically fragile cornea. However, even if there is a genetic support [6], no causative gene(s) has yet been found and most of keratoconus cases are sporadic. This multifactorial disease with underlying genetic, biomechanical, and environmental processes has remained poorly understood for more than 150 years. A high percentage of KC patients have an atopic disease or allergy, and as a consequence, they frequently rub their eyes [7]. Rabinowitz [8] and Naderan et al. [9] performed case-control studies which found that keratoconus patients rubbed their eyes more often than normal controls (80 and 83% vs. 58 and 52%, respectively).

Eye rubbing is of great concern to eye care practitioners in symptomatic patients, such as patients with ocular allergy, and they usually question patients about the frequency of these symptoms during their routine clinical practice [10]. Repetitive mechanical trauma can cause corneal weakening, by increasing apoptosis and oxidative damage, due to cyclic shear stress on corneal microstructures [11]. McMonnies and Boneham [12] proved a significant relationship between severe eye rubbing and keratoconus on the side of hand dominance. However, there are also examples in the literature [13] of keratoconus after chronic eye rubbing by the nondominant hand. The reported frequency of unilateral KC, as a subset of KC patients, varies depending on the methods used for diagnosis [14]. The reported frequency of unilateral KC using computerized videokeratography techniques ranges between 0.5 and 4% [2]. Different studies [15–19] have shown that KC patients display a higher prevalence of obstructive sleep apnea compared with the general population. Another study has shown an association between floppy eyelid syndrome and KC [20]; it seems to be a cause of corneal collagen crosslinking failure [21]. In our clinical practice, we have noticed that patients with unilateral keratoconus often sleep on their stomach or on their side, with a direct contact of the eye on the pillow (“pillow hugging”).

To our knowledge, there is no study in the literature that evaluates the possible influence of the patient’s sleep position on the development of keratoconus. Therefore, the aim of this case-control study was to assess eye rubbing and sleeping position in French patients with unilateral or highly asymmetric keratoconus (UHAKC).

## Methods

### Inclusion and exclusion criteria

This study included consecutive patients from the Rothschild Foundation, Paris, France, between January 2017 and April 2018. Patients with UHAKC were diagnosed by senior ophthalmologists in the team. Controls were subjects presenting to the preoperative refractive surgery clinic. All candidates for refractive surgery (controls) were myopic with or without astigmatism (Table 3). They were clinically and topographically evaluated and showed no signs of KC after examination by the ophthalmologists involved in this study. To maximize the power and comparison value of the study, the eye with the steepest keratometry was selected in control patients to be compared with the worse eye of the KC patients. The initial examination included measurement of best-corrected visual acuity (BCVA) as well as anterior segment, lens, vitreous, and retina evaluation with slit-lamp biomicroscopic examination. Both the Topolyzer (Oculus, Wetzlar, Germany) and Orbscan II topographers (Bausch & Lomb, New Jersey) were used to obtain maximum, minimum, and mean keratometry values (Kmax, Kmin, and Kmean, respectively) as well as the simulated keratometry (SimK) and central corneal thickness (CCT, μm), so that systematic errors due to keratometry measurements using different systems were therefore unlikely. The Ocular Responsible Analyzer ® (ORA; Reichert Ophthalmic Instruments, Buffalo, NY) system was used to evaluate corneal biomechanics (corneal hysteresis [CH] and corneal resistance factor [CRF]). A wavefront analysis was also performed using the OPD-SCAN III (Nidek Inc., Tokyo, Japan).

In order to classify a cornea as Normal (N), KC, or UHAKC (Table 1), we used a combination of quantitative videokeratography-derived indices [22] using different systems (Topolyzer and Orbscan). Normal eyes (N) were controls and UHAKC were cases. For the Orbscan, a Score Analyzer designed by Dr. D. Gatinel and Dr. A. Saad [23] was used. A SCORE (Score Analyzer, Orbscan) of > 4 in both eyes was chosen to define KC eyes; a SCORE of < 1.5 in both eyes was used to define normal eyes (controls); a SCORE of > 4 in the affected eye and < 1.5 in the contralateral eye was used to define UHAKC eyes. Based on the Topolyzer criteria [24–26], we used the Belin Ambrosio display final D index (BAD-D index) as this has shown better results than the surface curvature indices for detecting very mild forms of ectasia. In addition, patients in the KC group measured a BAD-D index (Topolyzer) > 5.0 in both eyes. The N group consisted of patients with a BAD-D index < 1.4 in both eyes. The UHAKC group measured a BAD-D index > 5.0 in the involved eye and < 1.4 in the contralateral eye. Regarding the OPD-SCAN III, the setting “keratoconus suspects (KCS) > 50%” was selected. In the UHAKC group, this criterion was present only in the affected eye and not in the contralateral eye. Worse eyes and better eyes were analyzed in UHAKC patients using topographic parameters such as the Kmax, Kmin, Kmean, CCT, and the inferior-superior value (I-S) in the 3-mm area. Exclusion criteria consisted of patients younger than 18 years old and older than 45 years old, patients with previous eye surgery or cross-linking, any compulsive disorders involving eye rubbing [27, 28] (autism, Tourette syndrome, Down syndrome), and the coexistence of an infectious or irritating ocular disorder (except allergy). The study was conducted in accordance with the tenets of the Declaration of Helsinki. Informed consent was obtained from all patients after presentation of the study protocol.

**Table 1:** Parameters used to define control eyes, keratoconus eyes and unilateral or highly asymmetric keratoconus (UHAKC) eyes

	Non-UHAKC eyes (N)	Keratoconus eyes (KC)	UHAKC eyes (UHAKC)
Orbscan II system (Bausch & Lomb)			
Score Analyzer > 4	No, in both eyes	Yes, in one or both eyes	Yes, in the affected eye
Score Analyzer <1.5	Yes, in both eyes	No, in both eyes	Yes, in contralateral eye
Pentacam (Oculus, Wetzlar)			
Belin/Ambrosio enhanced ectasia total derivation (BAD-D) > 5	No, in both eyes	Yes, in one or both eyes	Yes, in the affected eye
Belin/Ambrosio enhanced ectasia total derivation (BAD_D) < 1.4	Yes, in both eyes	No, in both eyes	Yes, in contralateral eye
OPD-SCAN III (Nidek)			
Keratoconus suspects (KCS) > 50%	No, in both eyes	Yes, in both eyes	Yes in the affected eye and not in the contralateral eye

### Questionnaire

We explained the goal of the research project to all patients that were invited to participate in the study. UHAKC and non-UHAKC patients were asked to complete a validated [29] self-administered questionnaire to collect exposure information anonymously (Supplementary data). The questionnaire included age, gender, ethnicity, family history of KC or corneal transplantation, health status, medications, allergies, and contact lens usage. We also interviewed a witness (the spouse or a family member) during the consultation, if present, with the patient’s agreement. In addition, subjects were asked to report their sleeping position (on the back, stomach, or sides), past and current ocular itching, as well as past and current eye rubbing. If “eye rubbing” was present, certain significant variables were ascertained. These included time and duration of eye rubbing, gentle or vigorous in nature, unilateral, or bilateral, and finally a demonstration was asked of the patient. At each follow-up visit, all UHAKC patients were reminded about the risk of continuing eye rubbing and the detrimental effects.

Additional informed consent was obtained from non-UHAKC patients in order to make available obscured (for anonymity) photos and/or videos of their eye rubbing and sleeping habits. This information is available at the website: https://defeatkeratoconus.com/allcases/ [30].

### Main outcome

The main outcome was the presence or the absence of independent predictors of the disease in the UHAKC group versus the control group. The topographic parameters and risk factors such as eye rubbing and sleeping position were also analyzed between the worse eye and the better eye in the UHAKC group only.

### Statistical analysis

A retrospective statistical analysis was performed by an independent statistician, using STATA Statistics® software. A *p* value < 0.05 was considered statistically significant. Cases and controls were individually only matched for age and sex. Normality of continuous data was assessed using the Anderson-Darling normality test calculator. A T test was used for continuous variables if the normality assumption was met and the Mann-Whitney test was used when the data were not normally distributed. Univariate and multivariate conditional logistic regression analyses were performed. Odds Ratios (OR) and 95% confidence intervals were calculated to determine whether any factor was significantly associated with unilateral KC. From a pilot study, a power calculation was performed, and the minimal sample size was estimated at 30 patients for UHAKC and 62 controls using the Kirkwood and Sterne formula, according to the “controls/UHAKC patients” ratio. Finally, we also performed a univariate analysis to compare worse eye and better eye in the UHAKC group.

## Results

### Demographics and corneal characteristics

Ninety-seven patients were included in this study: 33 cases (UHAKC) and 64 controls (normal).

Table 2 presents patient demographics in both groups. Cases and controls were matched for age and sex. The mean age was not significantly different between the UHAKC group and the control group (*p* = 0.45), 29.18 ± 7.38 years (range, 26–31 years) and 28.05 ± 6.33 years (range, 26–29 years), respectively. The mean follow-up duration was 20.3 months. In both groups, the majority of the participants were male (78.8% in cases group versus 78.1% in controls; *p* = 1). The BCVA was significantly lower in the UHAKC group (0.66) than in controls (*p* < 0.001). Most of patients were Caucasian and Arabic in the 2 groups; they were also Africans and more rarely Asians. There was no statistical difference for ethnicity, with a *p* value of 0.37 (see Table 2).

**Table 2:** Patient demographics in the UHAKC and control groups

	UHAKC	Controls (N)	*p* value
Total of patients	33	64	
Age (year)			0.45
Mean	29.18 (7.38)	28.05 (6.33)	
Range	26–31	26–29	
Sex			
Women	7 (21.2)	14 (21.9)	1
Men	26 (78.8)	50 (78.1)	
BCVA	0.66 (± 0.22)	1 (± 0)	< 0.001
Ethnicity (%)	Caucasian (52%), Arabic (33%), African (15%), Asian (1%)	Caucasian (59%), Arabic (25%), African (13%), Asian (3%)	0.37

BCVA: Best Corrected Visual Acuity

Table 3 shows corneal parameters for cases and controls Kmax, Kmin, and Kmean; Simulated keratometry (SimK) and Cylinder (Cyl) were significantly higher in the UHAKC group as compared to the control group (*p* < 0.001). In addition, CCT was significantly thinner in the UHAKC group as compared to the control group, 493.73 ± 31.07 μm versus 520.52 ± 22.69 μm (*p* < 0.001), respectively. Corneal hysteresis (CH) and corneal resistance factor (CRF) were significantly (*p* < 0.001) lower in the case group as compared to the control group. In contrast, the inferior-superior value (I-S) in the 3-mm area was significantly higher in the UHAKC group versus the control group, 1.66 ± 0.21 versus 0.47 ± 0.12 (*p* < 0.001), respectively. The mean refractive astigmatism was significantly higher in the UHAKC group versus the control group; − 1.82 ± 0.74 versus − 0.88 ± 0.65 (*p* < 0.001). The mean refractive sphere was not significantly different between groups.

**Table 3:** Corneal data in the UHAKC and control groups based on Orbscan values

	UHAKC eyes	Controls (N)	*p* value
Kmax (D)	45.78 ± 2.60	44.22 ± 1.33	< 0.001
Kmin (D)	44.07 ± 1.91	43.03 ± 1.23	< 0.001
Kmean (D)	44.94 ± 1.62	43.13 ± 1.03	< 0.001
SimK	2.13 ± 2.02	0.88 ± 0.67	< 0.001
Cyl (D)	1.87 ± 1.53	0.86 ± 0.59	< 0.001
CCT (μm)	495.73 ± 31.07	520.52 ± 22.69	< 0.001
CH	8.51 ± 0.49	11.30 ± 1.30	< 0.001
CRF	8.53 ± 1.63	12.18 ± 1.27	< 0.001
ISV (3 mm)	1.66 ± 0.21	0.47 ± 0.12	< 0.001
Mean Sphere	− 2.21 ± 1.01	− 2.6 ± 1.66	*p* = 0.08
Mean Astigmatism	− 1.82 ± 0.74	− 0.88 ± 0.65	< 0.001

*CCT* central corneal thickness, *CH* corneal hysteresis, *CRF* corneal resistance factor, *ISV* index of surface variance

### Univariate analysis

Table 4 shows the results of the univariate analysis of the various factors for the UHAKC and control groups. The strongest associations were found between the UHAKC group and eye rubbing during the day (OR = 172.78; *p* < 0.001) and sleeping on the steepest side (OR = 94.72; *p* < 0.001).

**Table 4:** Univariate analysis of risk factors for UHAKC

Risk factor	UHAKC eyes	Controls (N)	OR 95% CI	Pearson chi-square *p* value
*Family history of KC*			1.97 (0.12–32.51)	0.63
Yes	1 (3.0%)	1 (1.6%)		
*Eye rubbing during the day*			172.78 (21.13–1413.39)	< 0.001^*^
Yes	32 (97.0%)	10 (15.6%)		
*Eye rubbing in the morning*			24.3 (7.99–73.92)	< 0.001^*^
Yes	27 (81.8%)	10 (15.6%)		
*Eye rubbing on the steepest side*			21.7 (6.68–66.4)	< 0.001^*^
Yes	30 (91%)	7 (10.9%)		
*Eye rubbing with the dominant hand*			1.04 (0.29–3.73)	0.96
Yes	29 (87.9%)	56 (87.5%)		
*Allergy*			2.94 (1.15–7.48)	0.02^*^
Yes	25 (75.8%)	33 (51.6%)		
*Morning red eyes*			6.36 (2.52–16.01)	< 0.001^*^
Yes	23 (69.7%)	17 (26.6%)		
*Working at night*			0.87 (0.24–2.88)	0.87
Yes	20 (61%)	38 (59.4%)		
*Computer Work*			0.88 (0.34–2.30)	0.81
Yes	24 (72.7%)	48 (75.0%)		
*Stress at Work*			3.34 (1.39–8.03)	0.06
Yes	21 (63.6%)	22 (34.4%)		
*Sleeping on stomach or sides*			14.31 (4.78–42.84)	< 0.001^*^
Yes	28 (84.8%)	18 (28.1%)		
*Sleeping on steepest side*			94.72 (19.24–466.43)	< 0.001^*^
Yes	31 (93.9%)	9 (14.1%)		

*Indicates a significant risk factor (*p* < 0.05)

The recognized risk factors such as eye rubbing in the morning (OR = 24.3; *p* < 0.001), eye rubbing in the steepest side (OR = 21.7; *p* < 0.001), the presence of allergy (OR = 2.94; *p* = 0.02), red eyes in the morning (OR = 6.36; *p* < 0.001), and sleeping on stomach or sides (OR = 14.31; *p* < 0.001) were significantly associated with the presence of UHAKC in the univariate analysis.

The association between UHAKC and the stress at work was strong but not significant (OR = 3.34, *p* = 0.06). Family history of KC (OR = 1.97; *p* = 0.63), work at night (OR = 0.87; *p* = 0.87), working in front of a computer screen (OR = 0.88; *p* = 0.81) were not found to be significantly different between UHAKC and control group. Eye rubbing with the dominant hand was not significantly associated with UHAKC (OR = 1.04; *p* = 0.96).

### Multivariate analysis

Table 5 shows the multivariate analysis based on seven variables that were identified significant in the univariate analysis. The multivariate analysis confirmed the significance of five out of seven risk factors. Eye rubbing during the day (adjusted OR = 134.96; *p* = 0.002,) in the morning (adjusted OR = 24.86; *p* = 0.014), or in the steepest eye (adjusted OR = 27.56; *p* = 0.002) were significantly associated with the presence of UHAKC. Also, sleeping on stomach or sides (adjusted OR = 65.02; *p* = 0.001) or on the steepest side (adjusted OR = 144.01; *p* = 0.001) were associated with the presence of UHAKC. In contrast, the association between the presence of an allergy and red eyes in the morning was not confirmed in the multivariate analysis when all predictors were included in the model. The multivariate model containing these predictors was statistically significant (*n* = 97, df = 4, χ^2^ = 105.31, *p* < 0.001), indicating that it was able to distinguish between UHAKC and control patients.

**Table 5:** Multivariate analysis of risk factors for UHAKC

Risk factor	Adjusted OR	95% CI	*p*
Eye rubbing during the day	134.96	[6.35–2868.17]	0.002
Eye rubbing in the morning	24.86	[2.13–288.93]	0.014
Eye rubbing in the steepest side	27.56	[3.25–207.88]	0.002
Sleeping on stomach or sides	65.02	[5.05–573.91]	0.001
Sleeping on steepest side	144.01	[7.37–2815.88]	0.001

*Indicates a significant risk factor (*p* < 0.05)

### Analysis of the UHAKC group

Table 6 shows the corneal characteristics between the worse eye and the better eye in UHAKC patients, and the results of the univariate analysis of risk factors such as eye rubbing and sleeping position. Regarding topographic parameters, Kmax (*p* = 0.020), Kmin (*p* = 0.018), and Kmean (*p* = 0.036) were significantly higher in the worse eye than in the better eye. The CCT was significantly thinner in the worse eye than in the better eye, 495.73 ± 31.07 μm versus 509.14 ± 36.08 μm (*p* < 0.001), respectively. Similarly, the inferior-superior value (I-S) in the 3-mm area was significantly higher in the worse eye than in the better eye, 1.66 ± 0.21 versus 0.75 ± 0.16 (*p* < 0.001). The BCVA was also significantly lower in the worse eye (0.66 ± 0.22) than in the better eye (0.97 ± 0.10; *p* < 0.001). All patients in this group had a preference for rubbing their worse eye, and 90.9% were used to sleeping on the most affected side. A univariate analysis of eye rubbing and sleeping position was also performed to compare the worse eye and the better eye in the UHAKC group. Eye rubbing was 162.14-fold higher in the worse eye than in the better eye (*p* < 0.001). A higher number of patients were sleeping on the side of the worse eye than on the side of the better eye side (OR = 99.74; *p* < 0.001).

**Table 6:** Comparisons between the worse and better eyes in the UHAKC group

	Worse eye	Better eye	Pearson chi-square *p* value
*Parameters*			
Kmax (D)	45.78 ± 2.60	44.99 ± 1.82	0.020
Kmin (D)	44.07 ± 1.91	43.11 ± 1.71	0.018
Kmean (D)	44.94 ± 1.62	43.62 ± 1.65	0.036
CCT (μm)	495.73 ± 31.07	509.14 ± 36.08	< 0.001
ISV (3 mm)	1.66 ± 0.21	0.74 ± 0.16	< 0.001
BCVA	0.66 (± 0.22)	0.97 (± 0.10)	< 0.001
*Risk factors*			
Eye rubbing in the same side	33 (100%)	0 (0%)	*p* < 0.001; 162.14 [19.25–634.12]
Sleeping on the same side	30 (90.9%)	3 (9.1%)	*p* < 0.001; 99.74 [19.87–508.21]

## Discussion

This French case-control study aimed to identify new risk factors associated with UHAKC. The results showed a significant association between UHAKC and the following factors: eye rubbing, either during the day or in the morning and especially when it was done on the ipsilateral side; an incorrect sleeping position, i.e., sleeping on sides with the eye compressed against the pillow (“pillow hugging”) or on their stomach. Also, eye rubbing and sleeping on the side of the worse eye were significantly higher than the better eye side in UHAKC patients. This study was the first methodical analysis of an incorrect sleeping position and eye rubbing habits in unilateral or highly asymmetric KC. The results of this study regarding an association between eye rubbing and UHAKC were unequivocal. Our data also showed an association between an incorrect sleeping position and UHAKC, a finding that has never been described before in the literature, even if it had previously been suspected in patients with floppy eyelid syndrome [20, 31].

Eye rubbing has been shown to be associated with KC, and this was confirmed in our multivariate analysis. Although atopy and eye rubbing have been previously investigated [10, 32, 33], how eye rubbing is done and the sleeping position are new, especially in unilateral or highly asymmetrical KC, and are related to the focal nature. However, the cause of keratoconus is still unknown [34], but rubbing the eye is a well-known risk factor [34, 35]. This supports the hypothesis of mechanical fatigue of the cornea after repeated shear stress on its surface, and it could be a possible mechanism for initiating and/or inducing progression of KC, especially when the trauma is strong and prolonged. Korb et al. [36] have found that normal patients rubbed their eyes using their finger pads, generated a force < 0.45 kg/2.54 cm^2^, whereas KC patients used their knuckles, rubbing longer and more often, and generated a force > 4.5 kg/2.54 cm^2^. We agree that it is the more potent environmental factor for the development of KC [37]. In our study, all UHAKC patients were aware of the possible role of eye rubbing as a contributing factor in the development of KC, and after receiving this information, they controlled the impulse to rub their eyes. Some studies have already shown the important role of eye rubbing [26, 27, 34–36, 38–41], especially during adolescence.

For example, Coyle and al. [38] have reported the occurrence of unilateral KC in a boy who used digital massage of the affected eye to control episodes of paroxysmal atrial tachycardia. Additionally, Lindsay et al. [39] have reported the case of a patient with unilateral punctal agenesis and epiphora who developed unilateral KC due to regular eye rubbing and tear wiping.

Regarding the sleeping position, we were the first to identify it as a risk factor for KC. Interestingly, the most affected eye did not correlate with the dominant hand, but rather with the preferential side on which patients were used to sleeping. In our study, UHAKC patients were used to sleeping more often on the side of the worse eye than on the side of the better eye. Keratoconus appears to be more common in patients who sleep on their stomach or on one side, which results in a compression of the globe and could be related to increased heat generated locally during sleep. There are several mechanisms [40, 41] by which eye rubbing and the sleeping position could be involved in KC development. It could be assumed that the release of interleukin-1 by a damaged corneal epithelium could result in keratocyte apoptosis and tissue remodeling [42]. A mechanical trauma and increased intraocular pressure could also contribute to the pathogenesis. According to us, the association of all these environmental factors, including eye rubbing, allergy, and an incorrect sleeping position (ocular compression and increased temperature), could lead to the known biochemical changes in KC, i.e., increased activity of inflammatory mediators, induced keratocyte apoptosis, increased fibroblast activity, increased enzyme activities stretching the cornea (proteolytic enzymes) [43, 44], decreased proteinase inhibitor levels [45], and decreased stromal collagen. Thus, a vicious cycle could be induced because a biomechanical stress leads to biochemical changes and contributes to stromal thinning. Considering these arguments, we hypothesize that this focal biomechanical impairment could be primarily caused by eye rubbing, but probably with a stronger and prolonged impact of sleeping position in UHAKC eyes.

In our study, only one KC patient did not report any eye rubbing and no family history of KC, which is related to the fact that most KC cases are sporadic. Furthermore, compared with other studies, the higher percentage of eye rubbing in our study could be explained by the repetition of the question during each consultation, sometimes with the help of a relative. In some cases, patients initially denied rubbing their eyes because they were not aware that they do it automatically, and the testimony of relatives helped patients to realize that they do in fact rub their eyes.

Though not specified in our results, our study also conclude that there is no significant association between smoking and KC.

In addition to these considerations, patients with repeated episodes of allergy or dry eye could be at higher risk of keratoconus, again, due to excessive eye rubbing. Allergy has been found to be more prevalent in KC patients than in controls, as in the present study. The relationship between allergy and KC is less marked in our study than in other studies conducted in Israel [46], Lebanon [47], and Saudi Arabia [48]. This discrepancy may be due to the environmental influence of warm and sunny countries, which prevail in the abovementioned countries. This could explain the higher prevalence of KC reported in Middle East with an increased frequency of eye rubbing. We could consider the possibility that the direct and prolonged contact of the eyelids against the bed linen could increase the contamination of the ocular surface with irritants and allergens such as dust mites. This could contribute to increased local pruritus and subsequently to increased eye rubbing of the affected side, which could partly explain the asymmetry.

In our study, allergy was significantly associated with KC in the univariate analysis but not in the multivariate analysis. This could be due to the high prevalence of allergy in the control group (51.6%), which could be in turn due to the small sample size in this cohort.

In addition, we decided to study whether stress at work and working in front of a computer screen had any effect. We assumed that these factors could be responsible for eye rubbing and therefore could have participated in the asymmetric nature of KC. In our study, these items were not significantly associated with KC; again, this could be due to the small sample size and the high rates found in the control group. Thus, further studies are needed to confirm our findings.

Fortunately, not all eye rubbers or persons sleeping in an incorrect position will develop KC. Indeed, KC may well not develop before sustained and strong rubbing episodes after an extended period during which the cornea buckles permanently and induces irregular astigmatism and increased myopia via central or paracentral steepening. As already mentioned, we assumed that genetic factors for KC [49] could make the cornea more fragile and likely to develop KC, but in the absence of repeated and vigorous corneal trauma, KC does not tend to manifest. Patients with diseases that reduce corneal resistance could also be more likely to experience progressive and permanent corneal deformation whilst applying a similar eye rubbing trauma.

This study has some limitations. Most data were self-reported by patients so that they might be subjected to a recall bias or omission, but since both KC and control patients were asked the same questions, the relative difference should prevail. However, the questions were very similar to those used in the questionnaire validated in other studies [26, 29]. Another limitation is the controversial definition of unilateral or highly asymmetrical KC. In 2015, the Global Consensus on Keratoconus and Ectatic Diseases brought together 4 multinational corneal societies (Cornea Society, Asia Cornea Society, PanCornea and EuCornea) to establish consensus statements and recommendations for the diagnostic and managements of keratoconus. One of the conclusions of the consensus was that “true unilateral keratoconus does not exist” but, “secondary unilateral induced ectasia may be caused by a pure mechanical process” [14]. Tomography [29, 50] is currently recognized as the best and most widely used test to diagnose early or subclinical KC, and the use of quantitative videokeratography-derived indices [51, 52] could be more reproducible for quantifying unilateral or highly asymmetrical KC. Repetitive mechanical trauma [53] applied on the cornea results in a permanent thinning and deformation which also explains very well the frequent inter-eye asymmetry, as a large proportion of patients tend to rub one eye more than the other. We also evaluated the frequency of eye rubbing in the questionnaire, but we could not establish statistics on this item due to the variable and unreliable reports from patients. However, the importance of the frequency of eye rubbing is an important element in the development of keratoconus and tends to be associated with the severity and laterality of this disease. In some patients who rub only one eye, a strictly unilateral KC can be observed, and no clinically detectable topographic or biomechanical alteration can be found in the other unrubbed eye [54–56]. It should be noted that we monitored all patients of the UHAKC group for a minimum of 1 year, and none developed KC in the contralateral eye.

Furthermore, our relatively small sample size due to the low prevalence of unilateral KC and selective criteria used to define it limit the ability to discriminate with better precision, the risk among groups. A selection bias may have occurred in UHAKC patients because they were not aware that we compared them with non-UHAKC patients; the risk of systematic self-selection other than voluntary compliance is very limited. Therefore, cases and controls were individually matched only for age and sex, because it’s known that KC is more prevalent in men than women, and appears in the second and third decade of life. We decided to select candidates for refractive surgery as controls because most of them were young, healthy, with sufficient myopia, and/or astigmatism to perform refractive surgery, allowing groups to be compared after matching on age and sex. Finally, it is noteworthy that the obtained results were only based on a single dataset, which need to be confirmed by other studies. Nevertheless, our data on the prevalence of the known risk factors were very similar to published data.

In summary, our data provide strong evidence of an association between eye rubbing, incorrect sleeping position, and UHAKC, emphasizing the need for public health awareness of the deleterious consequences of vigorous eye rubbing and incorrect sleeping position. Medical colleagues need to improve the management of eye rubbing and to detect an incorrect sleeping position in diagnosed KC or patients at risk. Further studies are needed to evaluate the long-term stability of KC after eye rubbing cessation and adopting a new correct sleeping position.

## Electronic supplementary material

ESM 1 (DOCX 29 kb)

ESM 2 (PDF 168 kb)
