# Supplementary material for: Incorrect sleeping position and eye rubbing in patients with unilateral or highly asymmetric keratoconus: a case-control study
Source: Graefes Arch Clin Exp Ophthalmol. 2020 Jun 10;258(11):2431–9. doi: 10.1007/s00417-020-04771-z (PMC7584543; doi:10.1007/s00417-020-04771-z)
Supplement: Supplementary file 1 — (DOCX 29 kb) [file 417_2020_4771_MOESM1_ESM.docx]

**
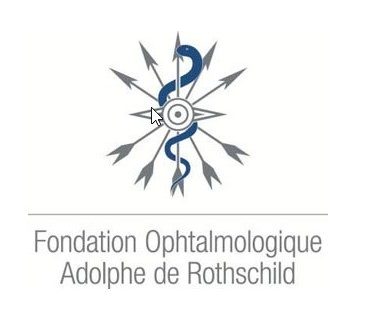
QUESTIONNAIRE : KERATOCONUS AND HABITS**

You are currently being followed at Rothschild Foundation, Paris for your eye disease, the keratoconus. The Keratoconus is a corneal disease, most often bilateral and asymmetrical. We are particularly interested in unilateral forms, and we are doing a study on it. For this we need to collect data from patients with unilateral keratoconus and also from healthy volunteers

The purpose of this questionnaire is to assess the importance of some habits like eye rubbing and sleep position in the development of keratoconus, particularly in unilateral forms.

Last Name : Age : Gender :

First Name : Date :

**1/ Do you used to rub your eyes during the day? :**

❒ Yes
❒ No

**2/ If yes, how often do you think you rub your eyes?**

❒ Rarely, not everyday
❒ Moderately, 1 time per day
❒ Oftenly, between 1 and 9 times a day
❒ Very often, more that 10 times a day

**3/ Do you preferentially rub your eyes in the morning ? :**

❒ Yes
❒ No

**4/ Which eye do you rub more frequently ?**

❒ Right
❒ Left

**5/ Why do you rub your eyes ?**❒ Itchy eyes
❒ Unconscious habits
❒ Dry eyes
❒ Ocular fatigue (screen work)

**6/ How often do those around you estimate your eye rubbing habit?**

❒ Rarely, not everyday
❒ Moderately, 1 time per day
❒ Oftenly, between 1 and 9 times a day
❒ Very often, more that 10 times a day

**7/ How often do those around you (family, spouses, friends) estimate your eye rubbing habit?**

❒ Rarely, not everyday
❒ Moderately, 1 time per day
❒ Oftenly, between 1 and 9 times a day
❒ Very often, more that 10 times a day

**8/ Do you have keratoconus history in your family ?**

❒ Yes
❒ No

**9/ Did you already corneal collagen cross-linking ?**

❒ Yes
❒ No

**10/ Which side is your dominant hand ?**

❒ Right
❒ Left

**11/ Do you have history of allergy ?**

❒ Yes
❒ No

**12/ Do you smoke ?**

❒ Yes
❒ No

**13/ Do you use to work at night ?**

❒ Yes
❒ No

**14/ Do you use oftenly your computer at work ?**

❒ Yes
❒ No

**15/ Are you oftenly stressed at work ?**

❒ Yes
❒ No

**16/ About your eyes, are they often red at the morning after wake-up ?**

❒ Yes
❒ No

**17/ Do you sleep with the eye buried in the pillow ?**

❒ No
❒ Yes
 ❒ Left side
 ❒ Right side

**18/ About your sleeping habit, you are used to :**

❒ sleep on back
❒ sleep on left side
❒ sleep on right side
❒ sleep on stomach

**Thank you for your participation**

❒ I give my consent to participate in this study

***Signature :***
