# Supplementary material for: Incorrect sleeping position and eye rubbing in patients with unilateral or highly asymmetric keratoconus: a case-control study
Source: Graefes Arch Clin Exp Ophthalmol. 2020 Jun 10;258(11):2431–9. doi: 10.1007/s00417-020-04771-z (PMC7584543; doi:10.1007/s00417-020-04771-z)
Supplement: Supplementary file 2 — (PDF 168 kb) [file 417_2020_4771_MOESM2_ESM.pdf]

Fondation Ophtalmologique Adolphe de Rothschild

QUESTIONNAIRE : KERATOCONUS AND HABITS

The Keratoconus Diary

You are currently being monitored at the Rothschild Foundation, Paris for your eye condition, known as keratoconus. Keratoconus is a corneal disease, most often bilateral and asymmetrical. We are particularly interested in studying unilateral forms of the disease, and are conducting research on it. For this we need to collect data from both, patients with unilateral keratoconus and healthy volunteers.

The purpose of this questionnaire is to assess the importance of some habits such as eye rubbing and sleep position in the development of keratoconus, particularly in unilateral forms.

Last Name : Age : Gender :

First Name : Date :

1/ Did you or do you rub your eyes during the day? :

❒ Yes
❒ No

2/ If yes, how often do you think you rub your eyes?

❒ Rarely, not every day
❒ Moderately, 1 time per day
❒ Often, between 1 and 9 times a day
❒ Very often, more that 10 times a day

3/ Do you preferentially rub your eyes in the morning ? :

❒ Yes
❒ No

4/ Which eye do you rub more frequently ?

❒ Right
❒ Left

5/ Why do you rub your eyes ?

❒ Itchy eyes
❒ Unconscious habit
❒ Dry eyes
❒ Ocular fatigue (screen work)

6/ How often do those around you (family, spouses, friends) estimate your eye rubbing habit?

❒ Rarely, not every day
❒ Moderately, 1 time per day
❒ Oftenly, between 1 and 9 times a day
❒ Very often, more that 10 times a day

7/ Do you have a history of keratoconus in your family ?

❒ Yes
❒ No

8/ Did you already have a procedure known as, corneal collagen cross-linking ?

❒ Yes
❒ No

9/ Which is your dominant hand ?

❒ Right
❒ Left

10/ Do you have a history of any allergies ?

❒ Yes
❒ No

11/ Do you smoke ?

❒ Yes
❒ No

12/ Have you currently or previously had a job that entailed night shifts ?

❒ Yes
❒ No

13/ Do you regularly use a computer at work ?

❒ Yes
❒ No

14/ Are you often stressed at work ?

❒ Yes
❒ No

15/ Regarding your eyes, are they often red in the morning when you wake-up ?

❒ Yes
❒ No

16/ Do you sleep with your eye buried in the pillow ?

❒ No
❒ Yes
  ❒ Left side
  ❒ Right side

17/ Regarding your sleeping habit, you normally :

❒ sleep on back
❒ sleep on left side
❒ sleep on right side
❒ sleep on stomach

Thank you for your participation

❒ <u>I give my consent to participate in this study</u>

Signature :
